# EVs and Bioengineering: From Cellular Products to Engineered Nanomachines

**DOI:** 10.3390/ijms21176048

**Published:** 2020-08-22

**Authors:** Simona Villata, Marta Canta, Valentina Cauda

**Affiliations:** Department of Applied Science and Technology, Politecnico di Torino, Corso Duca degli Abruzzi 24, 10129 Turin, Italy; simona.villata@studenti.polito.it (S.V.); marta.canta@polito.it (M.C.)

**Keywords:** extracellular vesicles, drug delivery, surface modification, cancer therapy, gene therapy

## Abstract

Extracellular vesicles (EVs) are natural carriers produced by many different cell types that have a plethora of functions and roles that are still under discovery. This review aims to be a compendium on the current advancement in terms of EV modifications and re-engineering, as well as their potential use in nanomedicine. In particular, the latest advancements on artificial EVs are discussed, with these being the frontier of nanomedicine-based therapeutics. The first part of this review gives an overview of the EVs naturally produced by cells and their extraction methods, focusing on the possibility to use them to carry desired cargo. The main issues for the production of the EV-based carriers are addressed, and several examples of the techniques used to upload the cargo are provided. The second part focuses on the engineered EVs, obtained through surface modification, both using direct and indirect methods, i.e., engineering of the parental cells. Several examples of the current literature are proposed to show the broad variety of engineered EVs produced thus far. In particular, we also report the possibility to engineer the parental cells to produce cargo-loaded EVs or EVs displaying specific surface markers. The third and last part focuses on the most recent advancements based on synthetic and chimeric EVs and the methods for their production. Both top-down or bottom-up techniques are analyzed, with many examples of applications.

## 1. Introduction

Extracellular vesicles (EVs) are nowadays well known as small vesicles [1] produced by almost all cell types, ensuring efficient communication among cells throughout the body by transporting lipids, proteins, and nucleic acids [2,3,4]. The presence of cell type-specific molecular signatures in these EVs has highlighted their potential role as biomarkers in a variety of diseases [2,3,4,5,6]. Moreover, there is huge interest in applying EVs or synthetic EV mimics in nanomedicine as drug delivery systems [4,7]. Their surface can be modified with various macromolecules, including peptides, dyes, or stealth polymers for diagnostic purposes; cell tracking; or even for targeting other cells or tissues [5]. EVs have shown to have a role in the immune system modulation or as promoters of carcinogenicity [4,5,8,9]. They have been recently shown to coat both organic and inorganic nanoparticles, thus producing novel biomimetic hybrids [6,9]. Therefore, they are becoming the invisible warriors of today’s nanomedicine, the camouflage suit for many devices and drugs [7,10,11,12,13]. 

The aim of this review is to give a deep overview of the EV-based solutions for nanomedicine. Firstly, we comment about the natural EVs, their biogenesis, their interactions with the cells thanks to their unique features, their isolation methods, the processes used to encapsulate a cargo into them, and their general applications. We then discuss some strategies used to engineer these extracellular vesicles in order to make them more specific and advanced. In particular, we focus on the functionalization of EVs’ surface with different molecules for various biomedical purposes, both with direct and indirect methods, and on the possibility to encapsulate cargo through the engineering of the parental cells. At the end, we look at the new frontier of EVs—the synthetic EVs, which are under study in order to have well characterized, reproducible, and scalable chimeric vesicles, containing only the necessary key elements for their specific purpose. In particular, we analyze both bottom-up and top-down techniques. 

Finally, we propose a clear and complete picture on the most interesting scientific efforts on EV usage and modification, their potential, and the possibility to customize them for a specific nanomedicine application. 

## 2. Natural EVs 

Every day, in the human body, cells release in the extracellular space particles delimited by a lipid bilayer that cannot replicate. Such particles are defined as extracellular vesicles (EVs) [8]. This general term encompasses a huge number of structures, referred as exosomes, microvesicles, microparticles, ectosomes, oncosomes, apoptotic bodies, and many other names [8], which differ in biogenesis, release pathways, size, content, and function. The classification of these EVs is a complex matter because they overlap in terms of many characteristics; thus, more than one parameter must be taken into account [8]. Furthermore, they are not associated to specific cells because every cell is able to release more than one EV type. 

**Figure 1:**
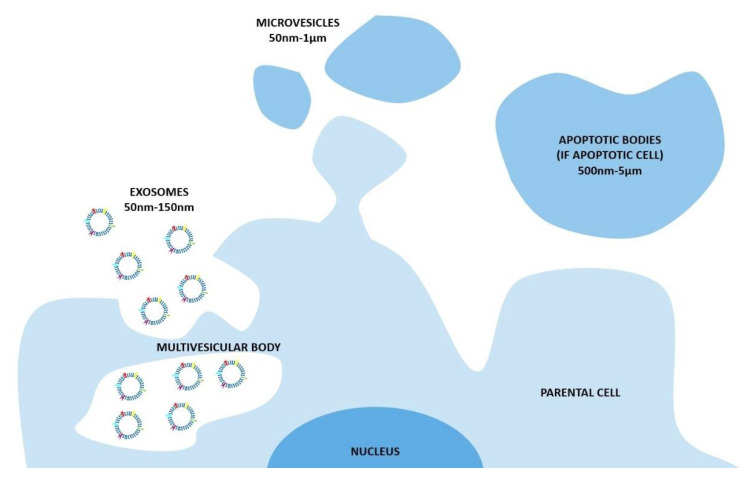
The biogenesis of extracellular vesicles (EVs) and the different pathways according to the current classification. In particular, exosomes consist of vesicles with an endocytic origin, ranging in size from around 50 to 150 nm. They originate as intraluminal vesicles (ILVs) of the multivesicular bodies (MVBs) and become exosomes when secreted in the extracellular milieu. The microvesicles originate from the direct outwards budding and fission of the plasma membrane and range in size from 50 nm to 1 μm. The apoptotic bodies are vesicles resulting from the disassembly of the apoptotic cells, which are generally defined as 500 nm-5 μm in diameter.

The nomenclature of these vesicles evolved during the last two decades [9]. The widespread and oldest classification divides the EVs on the base of their biogenetic pathway and, even simplistically, identifies three main classes: the exosomes, the microvesicles, and the apoptotic bodies (Figure 1). The exosomes consist of vesicles with an endocytic origin, ranging in size from around 50 to 150 nm. They originate as intraluminal vesicles (ILVs) of the multivesicular bodies (MVBs) and become exosomes when secreted in the extracellular milieu. The microvesicles originate from the direct outwards budding and fission of the plasma membrane and range in size from 50 nm to 1 μm, and in some case they can reach higher dimensions of up to 10 μm (this is the case with the large vesicles released by cancer cells, named oncosomes). Lastly the apoptotic bodies are vesicles resulting from the disassembly of the apoptotic cells, which are generally defined as 500 nm-5 μm in diameter [13]. 

In recent years, the International Society for Extracellular Vesicles proposed a new classification based on the size range [8]. In fact, as reported by Thery et al. [8], it is extraordinary difficult to assign an EV to a particular biogenesis pathway due to the lack of specific markers; therefore, a classification on a physical characteristic, such as the size, results as being most appropriate. In the most recent publications, EVs are divided into two main classes, defined as small EVs (<100 nm or <200 nm) and medium/large EVs (>200 nm).

### 2.1. EVs as Delivery Systems

The discovery of EVs is quite recent. Described for the first time in the late 1960s as “platelet dust” in the fresh plasma [14], for many years these vesicles were only marginally studied and only seen as a mechanism used by the cells to dispose of their cellular wastes. In fact, the explosion in EVs research took place in 2007 when the group of Valadi, from Goteberg University, published the article “Exosome-mediated transfer of mRNAs and microRNAs is a novel mechanism of genetic exchange between cells”, demonstrating that the EVs could be vectors of genetic information [10]. Indeed, despite some EVs serving to eliminate molecules from the cell [11], one of their main function is to be the mediator in the cellular communication. 

**Figure 2:**
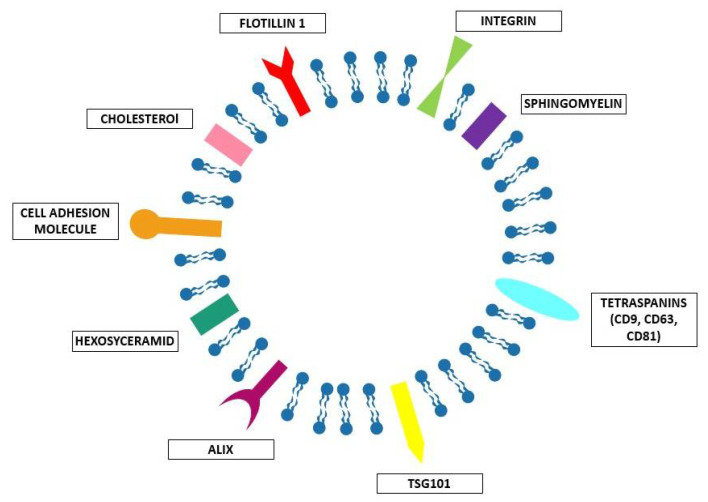
Schematic representation of EVs’ basic composition, including: integrins; tetraspanins such as CD9, CD63 and CD81; TSG101; Alix; cell adhesion molecule; flotillin1; cholesterol; sphingomyelin; and hexosyceramid.

Essentially, cells use different methods to communicate. When they are close to each other, they can exchange information through direct contact, using ligand-receptor signaling or transporting molecules and organelles across channels (gap junctions, microtubes). However, sometimes, they need to communicate between them in terms of packages of information, i.e., the EVs. Maas et al. [11] defined the EVs as a heterogeneous collection of membrane-bound carriers with complex cargoes including proteins, lipids, and nucleic acids. A typical EV structure is schematized in Figure 2. The EVs are composed of a lipid bilayer enriched by specific lipids such as cholesterol, sphingomyelin, and hexosyceramids [12], which confer stability to the vesicles and allow them to transport their cargo over short and long distances within the extracellular spaces and biofluids of the organism [13]. These lipid bilayers are often enriched by the so called EV-associated proteins, involved in cell recognition and binding, and also related to their biogenesis, such as TSG101 (Tumor susceptibility gene 101); Alix; flotillin 1; tetraspanins such as CD9, CD63, and CD81; integrins; and cell adhesion molecules [12]. However, the specific composition of each EV is extremely variable, including other specific proteins, nucleic acids, and sugars. This seems to reflect the composition and state of the parental cell, and as previously assessed [11] represents the “information” the EVs must deliver. Indeed, when the EVs are released in the extracellular space they are directed to specific target cells in order to deliver specific molecules. These molecules are associated with specific information and are able to trigger specific phenotypic changes in the receiving cells. It is easy to understand how the discovery of this peculiar EV function paved the way to their use as delivery systems for therapeutic purposes. However, as it will be discussed more in detail below in Section 2.1.2, the application of EVs as delivery systems requires a careful analysis of the kind of EVs to use in the specific systems, and many issues in EV-based targeted drug delivery are present, as has been recently described [15]. 

A myriad of publications has appeared in recent years that have aimed at the modification of the EVs for their loading with the desired cargo. The loading methods are numerous and will be analyzed in depth in the second part of this review. In fact, before starting the construction of the EV-based delivery systems, scientists must answer a range of different questions in order to solve fundamental aspects for the production of safe and reliable EV therapeutic tools. Essentially, as previously stated, cells also use different methods to communicate. When they are close to each other, they can exchange information through direct contact, using ligand-receptor signaling or transporting molecules and organelles across channels (gap junctions, microtubes) [16]. Cells also exchange information via tunneling nanotubes, which has synergies with EVs, as shown by Nawaz et al. [16].

#### 2.1.1. What Kind of EV–Cell Interactions Exist?

To exploit their role of cargo, the EVs must reach the desired target cells, release the cargo inside, and avoid undesired paths that could lead to cargo destruction or recycling. Indeed, the ways that EVs interact with the cells are varied, and a precise comprehension of the parameters determining the EVs’ fate is essential to exploit their function as cell to cell communication agents. 

**Figure 3:**
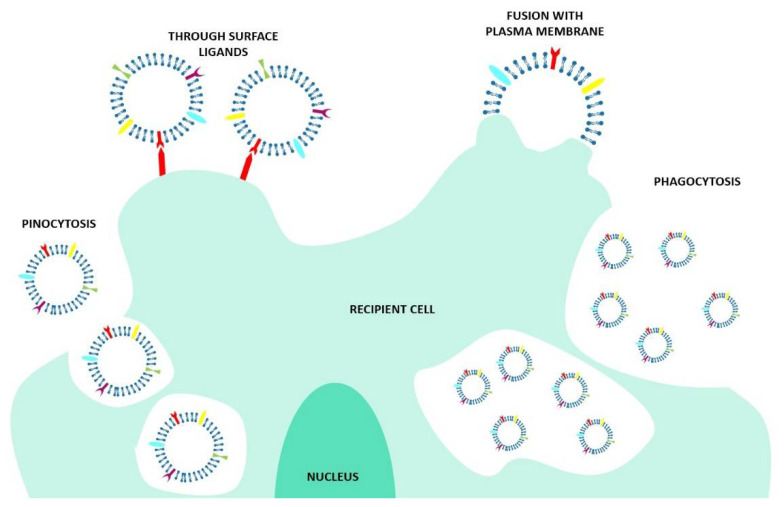
Schematic view of the mechanisms involved in the cellular uptake of EVs. In particular, EVs can interact with the cell through surface ligands, or they can be internalized through pinocytosis, phagocytosis, or fusing with the plasma membrane.

At present, the specific mechanisms of EV–cell interaction are not completely elucidated. The literature suggests that different cell types use specific pathways to promote EV entry into cells [11], but also the fact that one single cell could use more than one uptake mechanism for the different EVs [17]. Furthermore, the uptake could be performed in an unspecific way, such as through the micropinocytosis, but also could be triggered by specific interactions between receptors and ligands placed on both the EVs and the recipient cell membranes [18]. It has also been shown that EVs could be used for intra-organ communication [18]. For example, tumor-derived exosomes taken up by organ-specific cells are involved in the preparation of the pre-metastatic niche [19]. Almost all kinds of internalization mechanisms are reported in the literature, involving the fusion of the EV membrane with the recipient cell, multiple endocytosis routes (receptor-, clathrin-, dynamin-, and caveolae- dependent endocytosis), and also the pinocytosis and phagocytosis mechanisms, with the latter two being associated with EV clearance (Figure 3). 

In order to assess successful delivery of the desired cargo, it is important to understand the specific internalization mechanisms and EV fate. Indeed, while the fusion ensures the release of the EV content inside the cells, the endocytic pathways could result in cargo destruction inside the lysosomes or in cargo re-secretion in the extracellular space. Thus, as stressed from Mathieu et al. [16], to really assess the functional consequences of the EV-mediated cargo transfer, it is essential to investigate the internalization mechanism until the end point [17]. In addition, to add another layer of complexity, other mechanisms of information delivery, such as juxtacrine and soluble signaling, are not dependent on EV internalization. Both mechanisms induce the activation of cell signaling pathways after the activation of cell surface receptors that trigger a phenotypic change in the recipient cell. In particular, in the soluble signaling, the ligand expressed by the EVs is cleaved in correspondence with the cell surface, while in juxtacrine signaling, the juxtaposition of the EV ligands and the cell receptors takes place [20].

Lastly, in the design of an efficient EV carrier, it must be taken into account that the EVs not only interact with cells, but also with many components of the microenvironment they encounter during their “cargo transport”. Buzas et al. thoroughly investigated these kinds of interactions, reporting on the presence of incredible EV surface-associated molecules, such as coagulation factors, DNA, and enzymes. These molecules are able to establish contact with both the cells and the microenvironment, thus participating in a wide variety of biological processes and influencing the EV’s fate [21].

#### 2.1.2. Which Kind of Cells Produce EVs?

The idea of the use of naturally produced EVs as delivery agents was supported by their numerous advantageous characteristics that would potentially solve many problems associated with the existing nanodelivery systems [22]. Indeed, EVs present low immunogenicity [23], low cytotoxicity, and high biostability [24]; can load different cargos; and are able to target the recipient cells [4]. They are also biodegradable by cells and their clearance rate is lower than synthetic objects [25]. In fact, it has been demonstrated that the surface of some types of EVs is decorated by anti-phagocytosis surface markers, such as CD47, which work as a “don’t eat me” signal for the macrophages [26]. Another important skill is that EVs, as natural carriers, can cross barriers such as the blood–brain barrier, which are very difficult to overcome with bare drugs or other strategies [27].

However, despite these favorable characteristics, the application of EVs as delivery systems requires a careful analysis of the kind of the EVs to use in specific systems, with a particular evaluation of the EVs’ production source. 

Indeed, even if EVs are involved in a multitude of physiological roles, such as the control of cell homeostasis [28], it is known that they also participate in numerous pathological processes [29]. For instance, tumor-derived (TD) or tumor-associated (TA) EVs have been demonstrated to have a primary role in tumor progression and spreading, mediating a multitude of processes such as tumor invasion, drug resistance, angiogenesis, and immune escape [30,31]. Indeed, through the EVs, tumor cells not only communicate between themselves, but also with normal cells, modifying their phenotype or reeducating them to perform specific functions. TDEVs have been demonstrated to be able to drive the differentiation of normal fibroblasts of the tumor microenvironment into cancer-addicted fibroblasts (CAFs). CAF in turnsare able to secrete factors and EVs, promoting cancer proliferation, progression, invasion, and metastasis [32]. 

Considering this, it is easy to understand how the use of EVs produced by cancer cells as delivery agents must be carefully evaluated and adopted only after the neutralization of their pro-tumoral effects. At present, it seems that scientists are more prone to the use of these kinds of EVs as biomarkers for cancer diagnosis or in some applications of cancer immunotherapy than as delivery systems.

Despite this, the use of these EVs as carriers of therapeutic agents could offer some advantages—first of all, tumor tropism [33,34]. In fact, TDEVs could harbor an innate homing ability versus the parental tumor cells and the tumor microenvironment, thus exploiting a selective targeting for drug delivery in the tumor tissue [33,34]. In addition, some studies have showed that tumor cells produce a major amount of EVs compared to their normal counterparts, suggesting their major availability.

The use of EVs derived from normal cells bypasses all of these potential risks. However, it must be taken into account that the use of “normal”, but not autologous, EVs could result in unwanted immunogenic responses and adverse effects after administration. Even if a clarifying analysis of the EVs’ immunogenicity is still missing in the literature, a solution to this problem seems to come from the immature dendritic cells, reported to be immunologically inert and able to produce non-immunogenic EVs carriers [31,35].

Lastly, when deciding the EVs cell source, it must be considered that EVs produced by specific kinds of cells themselves harbor a therapeutic effect. For instance, mesenchymal stem cells (MSC)-derived EVs were demonstrated to harbor pro-regenerative and immunomodulatory properties, other than simply an anti-tumor effect [36]. In the same way, mature dendritic cell (DC)-derived EVs were demonstrated to be able to induce an anti-tumor response, maintaining the immunostimolatory properties of the parental cells [37]. These special characteristics make these cell lines special candidates for the creation of delivery systems that could combine the effects of the natural EV cargo with synthetically loaded cargo.

### 2.2. Method of EV Extraction 

Although this may appear trivial, the choice of EV isolation procedure is of primary importance for obtaining EVs intended for therapeutic purposes.

As described previously, the population of EVs encompasses a huge number of structures, each with particular features and functions determined by the lipids, nucleic acids, proteins, and sugars they are composed of and by which they transmit information [11]. In order to obtain a homogeneous EV preparation with specific characteristics and biological functions, scientists must not only standardize the culture conditions but also determine which extraction method allows for the better obtaining of the desired EV preparation. Indeed, several extraction methods have been developed, resulting in EV preparation that differ in terms of EV purity, yield, specificity, and quality, and that also demands different costs. 

**Figure 4:**
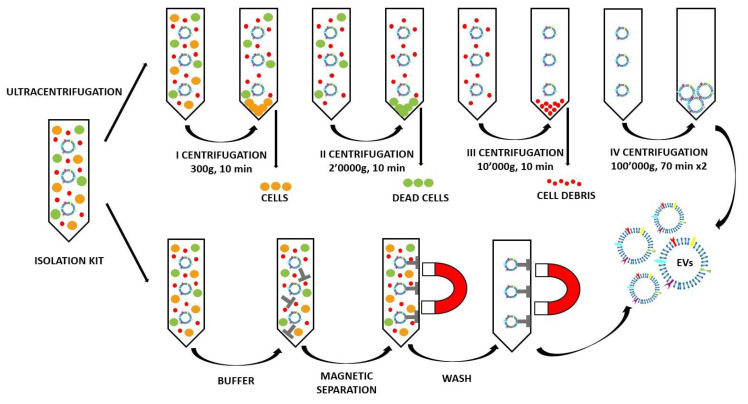
Example of EV isolation: differential ultracentrifugation with the various centrifugation steps from [38] and the isolation kit process.

The most commonly used and reported method to isolate the EVs is the differential centrifugation (ultracentrifugation) [38,39]. As shown in Figure 4 (top panel), in order to separate bigger objects from smaller ones (like the EVs), the EV-producing medium is subjected to a series of centrifugations with different speeds [38]. Larger particles sediment faster and are firstly removed, and thus the small EVs are found in the final pellet [38]. Although ultracentrifugation is the most widely used method nowadays, it harbors several drawbacks, such as EV aggregation and their possible damage due to the high speed [39]. However, the main caveat resides in the co-isolation of EVs with other small non-EV structures, such as exomers and high-density lipoproteins (HDL), which fall on the same size range [17,38,40].

The presence of these contaminants has a significant effect on the further analysis, also interfering with the correct EV identification [41,42] that is fundamental for the consequent clinical application of EVs.

A more purified preparation could be obtained through combining the size and density-based isolation methods. Indeed, the density gradient centrifugation allows for the obtaining of a pure EV preparation [43]. The EVs are separated on the basis of their size and mass density (top-down gradient) or mass density only (bottom-up gradient) by using gradient made by sucrose or iodixanol. However, despite the higher purity, this method is more laborious and time-consuming compared to the ultracentrifugation and results in low throughput, a main pitfall for EV clinical translation.

Other than the purity and yield, the scientist must consider the quality of the EV preparation in terms of specificity of the EV population. Different EV subpopulations could in fact harbor different functional properties that determine different biological fates. In fact, the separations based on size and density and also on the polymer precipitation (used by the majority of the isolation kits) do not allow for discrimination between the different EV subpopulations belonging to the same dimensional range. For this purpose, the purification methods based on the immunorecognition are considered the better choice. Indeed, the immunoaffinity methods use immobilized antibodies to selectively capture EVs. The antibodies could be linked to different supports, such as plates, columns, and magnetic beads, chosen to capture a specific EV subpopulation. As reported in Figure 4 in the lowest panel, in the isolation kit magnetic beads coupled with antibodies are used to recognize the EV surface antigens [44]. This method is highly specific and allows for the avoidance of any ultracentrifugation steps (and even centrifugation steps, as in the peculiar scheme reported in Figure 4) that could damage the EVs. However, in terms of its disadvantages, there is the possibility of an unsatisfactory release of the vesicles from the magnetic beads after separation and the low yield, which makes this method good for small volumes only [44,45,46]. 

### 2.3. Cargo-Loaded EVs

The natural role of EVs as carriers in cellular communication caused scientists to explore their potential role as therapeutic delivery systems [4]. Indeed, as previously discussed, EVs offer many advantages due to their intrinsic characteristics, such as their low immunogenicity and toxicity, biodegradability and biostability, possible intrinsic homing, and the ability to cross various body barriers. Furthermore, their unique structure, made of a hydrophobic lipid bilayer and a hydrophilic core, allows for the loading of a multitude of different cargoes. In essence, EVs have been reported to act as carriers of various molecules:

- Hydrophilic components such as hydrophilic drugs, but also microRNA (miRNA), small interfering RNA (siRNA), DNA, and proteins. They can be encapsulated in the hydrophilic core of the EV [47].
- Hydrophobic drugs, which can be incorporated in the lipid bilayer [47].
- Macromolecules for imaging, tracking (as fluorophore-conjugate antibodies), and targeting purposes. They can be bound with surface modifications to the EV lipid bilayers or surface proteins [47].

**Figure 5:**
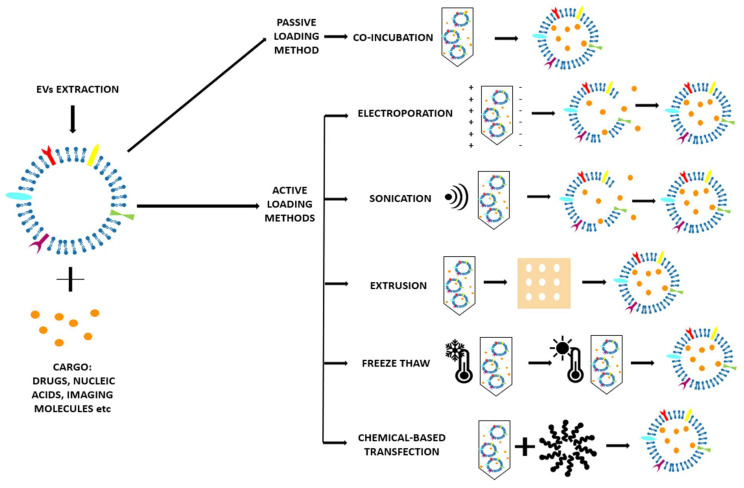
Scheme of the various loading methods with which it is possible to load EVs with the desired cargo. Specifically, the EV loading can follow two main approaches: the passive (co-incubation) and the active encapsulation (electroporation, sonication, extrusion, freeze–thaw, chemical-based transfection). The passive encapsulation is a relatively simple method in which the EV loading is obtained only through a co-incubation of the EVs and cargo, without the use of external stimuli. On the contrary, in the active encapsulation, the EVs are forced to load the cargo using many different strategies.

There are different EV loading strategies once they have been isolated, as reviewed in Table 1 and schematized in Figure 5. In particular, the EV loading can follow two main approaches: passive and the active encapsulation. Passive encapsulation is a relatively simple method in which the EV loading is obtained only through a co-incubation of the EVs and cargo, without the use of external stimuli. On the contrary, in the active encapsulation, the EVs are forced to load the cargo using many different strategies.

Each method harbors advantages and limitations and must be carefully evaluated in the specific context. Therefore, they will be discussed in detail in the following sections.

#### 2.3.1. Passive Loading Methods

##### Co-incubation

The cargo and the EVs are only incubated for a period of time at room temperature [51,54,55] or at 37 °C [48,49,53]. It is a very simple method, with the advantage of preserving the morphology of the EVs [5]. The cargo can diffuse into the EV following the concentration gradient and cross the membrane thanks to the small dimension and the lipophilic nature [48,49], or (as curcumin [54]) causing a lipid rearrangement of the membrane that facilitates the entry of the molecule. Other molecules, such as glucose, can be internalized by energy-dependent mechanisms (glucose channels) [53]. For the loading of nanoparticles, it is possible that the EVs adhere and then adsorb on the surface of the nanoparticles [52]. This method has, however, two main drawbacks: the low loading efficacy and the difficulty to assess the purity of the final preparation [26].

#### 2.3.2. Active Loading Methods

##### Electroporation

This consists in the application of an electrical field in a conductive (electrolyte) solution where the EV and cargo are dispersed. The electrical field creates temporary pores in the EV lipidic bilayer that allow the penetration of the cargo in the EVs [26]. This is important when large and hydrophilic molecules (for example siRNA [57] and miRNA) have to be incorporated, as they cannot diffuse through the membrane like the small hydrophobic molecules [55]. The main drawback of the electroporation method is the aggregation of EVs that must be limited through the optimization of the protocol [26].

##### Sonication

After EVs and cargo are mixed together in a water-based medium, they are sonicated with a homogenizer ultrasonic probe [49,51]. The cargo can penetrate because the shear forces from sonication induce the EV membrane deformation [26]. It seems that the integrity of the membrane can be restored; however, irreversible damage to EVs and possible aggregation can also take place [49].

##### Extrusion

After EVs and cargo are mixed together, they are loaded into a lipid extruder with a 100–400 nm porous membrane and are then extruded [51,55]. It is not yet clear how the membrane structure and properties are modified with this method [26].

##### Freeze–Thaw

This method provides repeated cycles of freezing at −196 °C in liquid nitrogen and thawing at room temperature of the solution of EVs and cargo [51]. The efficiency is higher than co-incubation method, but lower than the mechanical-based methods, such as sonication and extrusion [26]. Another drawback is that these freeze–thaw cycles can induce the aggregation of the EVs and modification of the membrane properties, i.e., protein orientation [5]. 

##### Chemical-Based Transfection

A surfactant is used to destabilize the membrane of the EVs to allow the penetration of the cargo [26]. Saponin is the most commonly used surfactant [51,55]; although guaranteeing good cargo loading efficacy into EVs, it could be toxic for living cells.

It is very interesting to notice that different loading methods were tried in certain studies in order to compare them and to find the best solution for the final application of EVs. In the study of [49], the authors performed co-incubation, electroporation, and sonication, finding that sonication gives the best results in terms of amount of loaded drug, followed by electroporation and co-incubation. They also demonstrated that the sonication process does not damage the protein and lipid structures of the EVs. Haney et al. [51] performed co-incubation, sonication, extrusion, freeze–thaw, and chemical-based transfection, finding that sonication, extrusion, and chemical-based transfection give the highest loading efficiency and a sustained release, while also proving the capability of these formulations for targeted delivery in vitro and in vivo. In the work of [55], Fuhrmann et al. performed co-incubation, electroporation, extrusion, and chemical-based transfection, finding that with chemical-based transfection, the loading of the drugs was up to 11-fold higher compared with the other methods tested (co-incubation, electroporation, and extrusion). The extruded EVs were demonstrated to cause cytotoxicity, whereas EVs loaded with the same cargo, porphyrin, but by co-incubation or electroporation did not show significant cytotoxicity. Finally, Shtam et al. [57] performed both electroporation and chemical-based transfection. They found that while chemical-based transfection was inapplicable for their purpose, electroporation, after the optimization of the parameters, was successful at introducing the heterologous siRNAs into the exosomes.

## 3. Engineered EVs 

After their isolation, EVs can be modified in order to obtain enhanced targeting and biomimetic features [5]. This concept is called engineering of EVs because, starting from naturally-derived EVs, scientists produce a vesicle with the desired behaviour [26]. It is important to highlight that an extracellular vesicle can be modified through both acting on the parental cells (indirect method) and by directly modifying the vesicle once it has been isolated (direct method) [5]. Another important branch of EV engineering is their hybridization after their isolation, where EV membranes are fused with synthetic liposomes [59].

### 3.1. Indirect Methods

**Figure 6:**
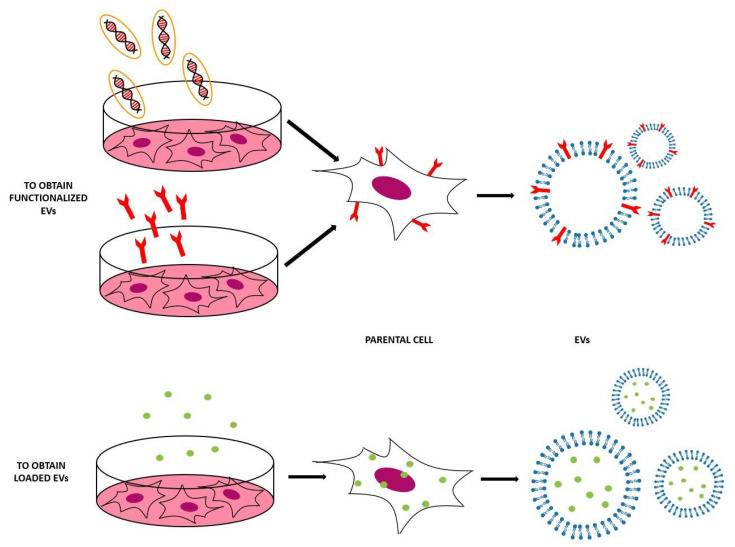
Scheme of the indirect methods used to engineer the EVs, both to functionalize EVs with the molecules of interest (to obtain EVs that expose these molecules on their surface) and to obtain EVs loaded with the desired cargo.

This method is based on the engineering of parental cells, i.e., the cells that will produce the EVs [59]. First, parental cells can be genetically or metabolically modified to alter the surface expression of the produced EVs and thus enhance their targeting ability and biocompatibility [5]. This can be carried out by inserting the coding sequence of the ligand of interest inframe to the coding sequences between the signal peptide and N-terminus of the mature peptide of a transmembrane protein [26]. Using a retrovirus or a lentivirus as gene transfer vector, this package is transmitted and expressed in parental cells [59]. At this point, these transfected parental cells will produce EVs with the desired peptide expressed on their surface. In Table 2 and Figure 6, some applications of this indirect method are reported [5,59]. 

Secondly, parental cells can be incubated with drugs or drug-loaded (or even gene-loaded) nanoparticles (NPs) in a sublethal concentration [5]—after a certain period of time, the therapeutic molecules or NPs will be internalized into the cells and then these cells will produce EVs containing a certain fraction of drug or drug-loaded NPs [5]. In this case, the loading of the cargo is obtained through the engineering of the parental cells [26]. For example, mesenchymal stromal cells (MSCs) can acquire strong anti-tumor activity after priming with paclitaxel (PTX) because MSCs secrete a high amount of membrane microvesicles that will contain the drug [75]. Another study reported how melanoma cells can be loaded with survivin T34A and gemcitabine to produce exosomes that carry the drug to treat pancreatic adenocarcinoma [76]. Doxorubicin and methotrexate have been loaded into tumoral cells and their apoptotic bodies containing the drug have been used to kill tumor cells, with reduced side effects [77]. Cells have been loaded with NPs also— superparamagnetic iron oxide nanoparticles (SPIONs) have been loaded in mesenchymal stem cells to produce charged EVs to treat leukemia [76], while iron oxide NPs and a photosensitizer have been encapsulated in HUVECs and human macrophages to obtain EVs to treat prostate and cervical cancer, respectively [78,79]. Gene therapy can also be carried out with this approach—for example, mesenchymal stem cells have been loaded with different miRNAs to obtain EVs [80]. The purpose of these EVs were varied, i.e., to increase sensitivity of tumor cells to chemotherapeutic drugs (miRNA-122 [81]), to inhibit the migration of osteosarcoma cells with miRNA-143 [82], and finally to inhibit glioma growth with miRNA-146b [83]. Moreover, chemically modified exogenous mRNA can be loaded in this way into EVs to produce a protein of interest [84].

It is important to focus not only on the technical challenges of producing engineered EVs with indirect methods, but also on the various biological issues that are concerned before, during, and after EV engineering. As a preliminary step before the engineering process, it is important to design the engineered EVs and to make the right choice in terms of parental cells. Many authors decided to use cell lines such as endothelial cell lines (HUVECs) [71,72] or dendritic cells (DCs) [62,65,67], while others worked with more tissue-specific cell lines. From the literature, it is evident that the main challenge in the choice of the parental cells is to become able to work with a patient’s derived cells in a controllable way and with introducing scalable protocols. For example, one of the critical issues is to obtain EVs with characteristics compatible to the cells with which they will interact. During the engineering, it is important to choose the proper surface modification to achieve the purpose and also to pay attention to the possible unwanted effects. Another challenge is to identify the most efficient way to obtain the functionalization. One of the most popular choices is to transfect the parental cells with the right plasmid vectors and their building is nowadays an important investigation subject in the biological field [60,61,62,63,64,65,66,67,74]. The other popular approach is to incubate the cells with DSPE-PEG (1,2-distearoyl-sn-glycero-3-phosphoethanolamine-polyethyleneglycol) to both link and further space the membrane from the targeting molecules. Such functional lipids can be actually bound to targeting ligands such as biotin, folate, thiol groups, or arginylglycylaspartic acid (RGD). Biotin can in turn selectively bind to streptavidin, being used for further functionalization [70,71,72]. Folate is able to target specific cancer cells [69,70], while thiol groups are useful in many binding reactions [68]. RGD is one of the most common sequences of cellular attachment at the extracellular matrix [68]. 

After the functionalization, the main biological challenge is to choose the most appropriate cell line or animal model to test the engineered EVs. One of the most popular choices is to use immortalized cell lines, for example HeLa [63,68,69], 3T3 [70] and Neuro2A [3,61], due to their advantages in terms of cost, ease of use, and ethical concerns. Indeed, even if not specific like the primary cell lines, they allow for the ability to overcome the main biological challenges of EV testing, such as it being time-consuming and having scalability issues, thus allowing movement from in vitro to in vivo testing easily.

Most of the authors that tested their formulation in vivo chose transgenic [64,65] or non-transgenic mice that bear [68,71,72] or do not bear [61,62,66,67,69,70] autologous tumor or xenografts and that could be athymic [73] or not. Unfortunately, these animal models are not complex enough to simulate the human system, and thus more investigation efforts must be pursued to develop more appropriate testing platforms. 

### 3.2. Direct Methods 

**Figure 7:**
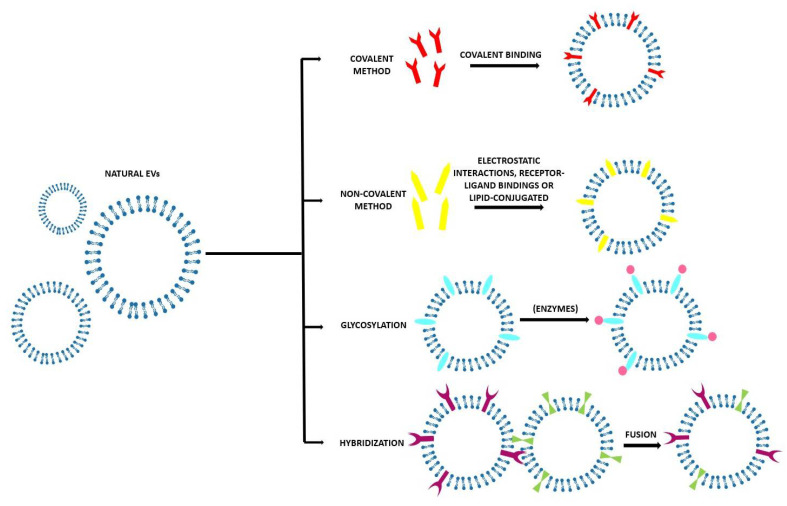
Scheme of the various direct methods to obtain engineered EVs with the desired characteristics and with the molecules of interest on the surface. In particular, covalent method; non-covalent methods such as electrostatic interaction; receptor–ligand binding; or lipid conjugation, glycosylation, or hybridization.

Several methods are used to modify the surface of EVs after their isolation. These modifications can be carried out to achieve more specific targeting or mimetic features [59]. Most frequently, the aim is to obtain fluorescent and magnetic labelling to track EVs, their biodistribution, and their pharmacokinetics to investigate their possible diagnostic and therapeutic applications [5]. As EVs are very delicate, it is necessary to pay attention to the reaction conditions to avoid their disruption and aggregation due to inappropriate temperature, pressure, and osmotic stresses [26]. Working in mild conditions can help to obtain the most controlled results [5]. After their isolation, EVs’ surfaces can be modified in different ways, as reported in Table 3 and Figure 7. 

#### 3.2.1. Covalent Methods

As the classical crosslinking is not enough in terms of specificity and efficiency, the most used covalent method nowadays is the Click Chemistry approach, also known as azide alkyne cycloaddition [103]. With this process, an alkyne moiety reacts with an azide group to form a stable triazole linkage [103]. Some studies also used a copper catalyst to accelerate the reaction [104], but several authors demonstrated that a successful binding can be obtained also without the copper catalyst [105]. One of the strengths of this method is that the experimental conditions are mild and that it can take place in both in organic and aqueous media (water, alcohols, dimethyl sulfoxide (DMSO)) [106]. The yield is high, the method is simple, and it does not impact on EV size nor on the target cell uptake [106]. This method does have, however, some drawbacks—the alkyne modification of the EV surface most likely occurs on the amine groups of the proteins instead of those of the phospholipids, introducing the possibility that the EV protein function may be inhibited [103]. By controlling the number of alkyne groups, it is possible to avoid the over modification of EV membrane proteins—with a standard calibration curve it has been estimated that approximately 1.5 alkyne modifications are made for every 150 kDa of EV protein [86]. A very common approach is PEGylation, the modification of EVs’ surfaces with polyethylene glycol to extend the circulation half time of the EVs. The drawback of PEGylation is that the PEG corona also reduces the EV–cell interaction and the cellular uptake of the EVs [87]. This disadvantage can be overcome by functionalizing the distal end of the PEG chain with a targeting ligand [5]. 

#### 3.2.2. Non-Covalent Methods

These methods are based on mild reactions, such as electrostatic interactions, receptor–ligand bindings, and lipid-conjugated compounds post-insertion into the EV’s lipid bilayer [5]. Electrostatic approaches usually involve highly cationic species adhering on negatively charged functional groups present on the biological membranes [5]. A possible drawback of these methods is that certain cationic nanomaterials can cause cytotoxicity and that they are typically taken up into the cells via endocytosis, leading to lysosomal degradation [26].

#### 3.2.3. Glycosylation 

Glycosylation is at the base of many biological functions of EVs, such as cargo protein recruitment and cellular recognition and uptake [107,108]. Alterations in the glycosylation pattern has been associated with different pathologies, for example, cancer, and these changes are closely correlated with the specific malignant transformation and progression. This evidence has led to make glycan structure a useful target for anti-tumor applications in theranostics [109,110]. The manipulation of glycosylation can be done using either enzymes or not.

#### 3.2.4. Hybridization

This method implies the fusion of natural EVs with their artificial counterpart, liposomes, to optimize the properties of native EVs [26]. This can be obtained thanks to the lipid composition of the EV membrane. In this way, the colloidal stability of EVs is improved, increasing their half-life in blood and modifying their immunogenicity profile, possibly decreasing it [103]. The lipid composition has been evidenced to impact on the cellular uptake—EVs hybridized with neutral or anionic lipids have a higher possibility to be taken up by cells than those hybridized with cationic lipids [103]. Moreover, hybridization of EVs increases the vesicle size (in a technique-dependent way)—this is a drawback because it decreases the in vivo retention of the vesicles, but also an advantage as it can improve the drug encapsulation efficiency [103]. Native EVs are actually very small in size and thus limited in their ability to encapsulate large molecules, while larger hybridized EVs can carry larger cargos [103].

As for the indirect methods, it is important to remember that the technical challenges to engineer the EVs with the different direct methods are directly correlated with the biological challenges that are fundamental in every step of EV engineering, from the preliminary design to the real environment testing. For what concerns the choice of the parental cells, in some works the authors chose the RAW 264.7 macrophages, an immortalized cancer cell line [89,91,100], while others used immortalized cell lines such as HeLa [92] or Neuro 2A [87], or even extracted the desired cells directly from mice [89] or human serum [94]. As stated previously, the main biological challenge is to find a scalable and controllable way to use the patient’s cells as source in order to obtain EVs that are possibly compatible to the patient environment.

As for the cargo loading, the best EV engineering method must be carefully evaluated in a specific context, considering advantages and limitations. In particular, for what concerns the functionalization, it is important to find the proper molecule for the desired purpose, and a variety of functionalizations are reported in the literature, as mentioned above. As for the indirect methods, the use of DSPE-PEG [88,89,100] or DMPE-PEG [87], as spacer to expose the functionalization, is a commonly used strategy. Finally, for both in vitro and in vivo testing steps, the biological challenges are the same listed above and analyzed for the indirect methods in terms of choice of the best cell line and/or animal model.

At this point, it is clear that the functionalization of EVs with ligands and other molecules can boost up their stability in blood circulation, have the capability of localizing the target site, and can increase their intracellular delivery efficiency [111]. The main drawback of EV engineering is the introduction of the risks of altering the orientation of membrane proteins, which may compromise their biological functionalities or even induce immunogenicity [59]. Further risks of EV engineering are associated with the hiding of these proteins or to the damage or disruption the EV membrane [101]. For this reason, developing bioinspired, synthetic, and chimeric EV-like alternatives is increasingly promising to broaden the therapeutic application of these natural biovesicles [111].

## 4. Synthetic and Chimeric EVs

As stated previously, EV-based nanomedicine has many advantages, such as the specificity in targeting and innate biomimicry. However, this approach has severe drawbacks too, such as the lack of purification protocols at a large-scale clinical grade, the potential safety concerns, the parental cell-dependent composition, and the inefficient drug payload [111]. These reasons are keeping EVs far from becoming a therapeutic reality [111]. To overcome these drawbacks, some alternative strategies have been promoted to develop artificial EVs. To build these particular devices, two main methods have been developed: the top-down and the bottom-up approaches.

### 4.1. Top-Down Approaches

The top-down method is based on the disruption of the cells of interest in little fragments that will then self-assemble in nanovesicles and microvesicles of various sizes with the same membrane features of the initial cell. The breaking of the cell membrane is physically obtained and for this reason these vesicles are also called physical-origin EVs. As they are obtained from cells, they are a good imitation of EVs and they incorporate the proteins and the biologically active molecules, but the yield of the production can increase by 100 times [112]. These artificial EVs can be obtained in two different ways—the simplest is extruding the cells through polycarbonate membranes with decreasing pore size (for example, from 10 µm to 5 µm to 1 µm [112]). The choice of the pore size is important—in this way, the cells, which are typically bigger than 10 µm, are disrupted by the first membrane, while organelles bigger than 1 µm are retained by the last membrane. The obtained vesicles thus have a diameter in the range below 1 µm. 

The other top-down method consists of using a microfluidic device that contains an array of hydrophilic microchannels. After injecting the cells in this device, they undergo to the shear-stress and they break in the membrane fragments that will then reassemble mainly in nanovesicles. One of the main advantages of top-down methods is that the techniques to modify cells before EV isolation can be easily applied, in this case in order to obtain specific components on the artificial EV membrane [113]. As the nanovesicles are directly derived from cells, they have a high biocompatibility, reduced clearance, and enhanced delivery efficacy thanks to the increased cellular uptake. These nanovesicles can be used to carry cargos or as therapeutic agents (without cargos) for cancer immunotherapy [114], cell proliferation, and tissue regeneration [115]. The top-down approach has disadvantages too—it is necessary (as for natural EVs) to have a purification protocol and it is very difficult to control the production and standardize the properties of these artificial EVs. The biological challenges that are directly linked with the top-down method in terms of parental cell choice and in vitro and in vivo testing are the same listed for the engineered EVs. 

**Figure 8:**
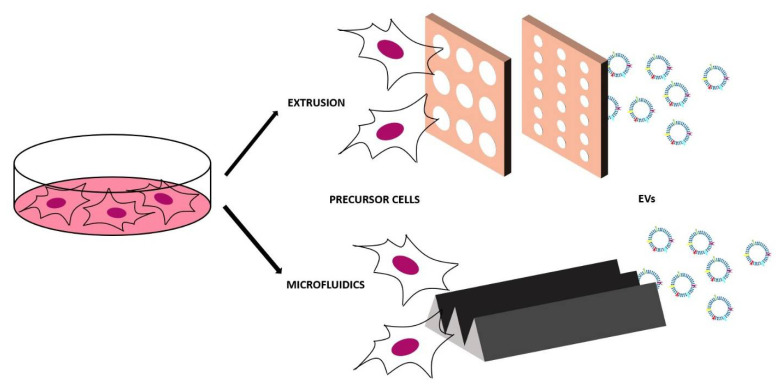
Scheme of the two main top-down approaches to produce synthetic EVs: extrusion and microfluidics. Top-down approach is based on the disruption of the cells of interest in little fragments that will then self-assemble in nanovesicles and microvesicles of various sizes with the same membrane features of the initial cell.

Some examples of top-down approaches are reported in Table 4 and Figure 8.

### 4.2. Bottom-Up Approach

The bottom-up method starts from small components, i.e., molecular building blocks to obtain complex structures, namely, the synthetic EVs [111,113]. The aim is to mimic the natural EVs using specific lipid composition and then functionalize this synthetic lipid bilayer (liposome) with the proteins that are necessary for targeting/biomimetic purposes with the same techniques used to engineer the natural EVs [113]. This method was developed through starting from two important hypotheses:

^1.^  Not all the components in natural EVs are essential for the specific therapeutic application [111].
^2.^  Liposomes have a spherical lipid bilayer structure, as the EVs and their properties, such as diameter, lipid composition, and functionalization, can be tuned [113].

To obtain the starting liposome, two main techniques are applied [113]—the simplest is the thin film hydration in which a dried film of lipids is hydrated by an aqueous medium containing the desired cargo [113]. The other one is based on a microemulsion approach and on a micelle assembly in the medium containing the compound to be encapsulated [113]. Both methods present the advantage to produce fully artificial EVs, with the wanted clean composition, scalable production protocol, and the use of pharmaceutical acceptable components that make bottom-up EVs a high pharmaceutical grade product [113]. However, bottom-up methods also have drawbacks—it is necessary to have a deep knowledge of every EV component in order to understand how to build a synthetic one; the high-purity lipids are often very expensive and it is possible that the proteins lose their function during the process [111]. 

**Figure 9:**
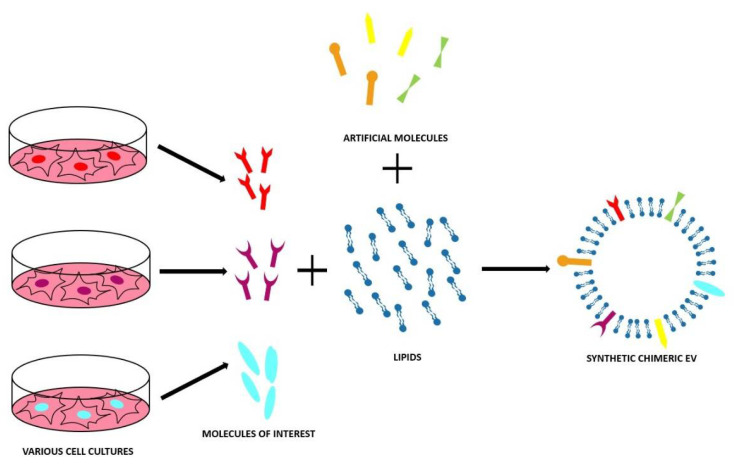
Scheme of the bottom-up approach to obtain synthetic chimeric EVs using artificial compounds or molecules from cells. The bottom-up method starts from small components, i.e., molecular building blocks to obtain complex structures, namely, the synthetic EVs. The aim is to mimic the natural EVs using specific lipid composition and then functionalize this synthetic lipid bilayer (liposome) with the proteins that are necessary for the targeting/biomimetic purposes with the same techniques used to engineer the natural EVs.

Examples of bottom-up approaches are reported in Table 5 and Figure 9.

## 5. Conclusions and Future Perspectives 

EVs are nowadays considered one of the main actors in the nanomedicine scene, thanks to their incredible features in terms of biocompatibility, cargo loading, cellular uptake, and immune system escaping. A step forward was made when researchers started to customize these exceptional nanovectors, engineering their surface with specific biomolecules for different purposes, such as tracking in vivo or targeting the desired cell type. Unfortunately, the EVs that are naturally derived by cells and engineered EVs have to fight against the current lack of reliable purification protocols, the difficulty of producing EVs with a controllable composition, and the inefficient cargo-loading capacity, which are the main obstacles in terms of a scalable clinical application of EVs. For what concerns the engineered EVs, another obstacle is the removal of the uncoupled molecules or cargo (such as drugs or nanoparticles) after the coupling process in order to purify the obtained formulation. For these reasons, some researchers decided to change the route by starting to produce new synthetic or chimeric EVs in order to obtain nanovectors that contain only the components that have been proven to be useful for the formulation purpose, inserting them in synthetic lipid nanovesicles. The future perspective of this research is the formulation of clean, purified, highly controllable, and pharmaceutically acceptable EV-like nanovesicles, which will allow researchers to abandon the difficult and minimally controllable protocols of EV extraction and selection. The main obstacle to achieve this step is the need to identify the components of the EVs that are fundamental to having a therapeutic and targeting effect, to understand how they behave if introduced in a synthetic lipid membrane, and to develop protocols to isolate and purify them in a scalable and controllable way. Of course, these challenges have to be faced up by multidisciplinary collaborations, which include biologists, chemists, material scientists, and engineers.

## 6. Patents

“Biomimetic Non-Immunogenic Nanoassembly for the Antitumor Therapy” WO2019/092550, priority on 13 November 2017. Inventors: V. Cauda, G. Canavese, T. Limongi, N. Garino, M. Laurenti, B. Dumontel, M. Canta, L. Racca, and A. Ancona of Politecnico di Torino.

“A biomimetic nanoporous carrier comprising an inhibitor directed towards the native form of IDH2 protein” IB2020/050401, priority on 23 January 2019. Inventors: V. Cauda, T. Limongi, L. Racca, M. Canta, F. Susa, R. Piva, E. Bergaggio, N. Vitale, and E. Mereu of Politecnico di Torino and Università degli Studi di Torino.

## Figures and Tables

**Table 1:** The various EV loading methods.

Parental Cells	Cargo	Loading Conditions	Recipient Cells	Treatment Condition	Application	Reference
**Co-Incubation**						
H1299 and YRC9	Doxorubicin conjugated with gold NPs	Incubated at 37 °C with 250 rpm for 2 h	H1299, A549, MRC9, and Dox-sensitive HCASM	1 × 10^5^ cells per well and EVs with the equivalent of 5 μg Dox per well	Anticancer activity against human lung cancer cells	[48]
RAW 264.7	Paclitaxel	Incubated at 37 °C for 1 hour with shaking	MDCK_WT_, MDCKMDR1, and 3LL-M27 *IN VIVO:* C57BL/6 mice	5000 cells per well and exosomes *IN VIVO:* i.n. 10^7^ particles/10 μL × 2	Overcome multiple drug resistance in cancer cells	[49]
KB	ZnO nanocrystals	Various loading conditions	KB	3 × 10^4^ cells per well and EVs with the equivalent of 15 μg/mL of ZnO nanocrystals	Treatment of cancer cells	[50]
RAW 264.7	Enzyme catalase	Incubated at RT for 18 hours	PC12 *IN VIVO*: C57BL/6 female mice	50,000 cells per well and EVs 230 µg total protein/mL *IN VIVO:* i.n. or i.v. 2.4 × 10^10^ EVs	Parkinson’s disease therapy	[51]
HeLa	MOF loaded with calcein	Incubated at 37 °C for 1.5 h with shaking	HeLa	1000 cells for each EV concentration (10−140 μg/mL)	Efficient drug delivery platform	[52]
MSCs	Glucose-coated gold NPs	Incubated for 3 h at 37 °C	*IN VIVO*: C57bl/6 male mice	*IN VIVO*: i.n. and i.v. 2.8 × 10^9^ EVs	In vivo neuroimaging	[53]
EL-4, MDA-MB231, 4T-1	Curcumin	Mixed at 22 °C, then sucrose gradient centrifugation	RAW 264.7 *IN VIVO*: 7- to 10-week female C57BL/6j mice	Exosomal curcumin 20 µmol/l, LPS 50 ng/mL *IN VIVO:* i.p. 4 mg/kg exosomal curcumin, 18.75 mg/kg LPS	Deliver anti-inflammatory agents to activated myeloid cells in vivo	[54]
MDAs, hUVECs, hMSCs and hESCs	Porphyrins of different hydrophobicities	Incubated at RT for 10 min	MDA-MB231	20,000 cells per well and EVs diluted 1:2 from the Stock solution (1.5 mg/mL of Por)	Improve the cellular uptake and photodynamic effect of porphyrins	[55]
**Electroporation**						
RAW 264.7	Paclitaxel	1000 kV for 5 ms, then incubated at 37 °C for 30 min	MDCK_WT_, MDCKMDR1 and 3LL-M27	5000 cells per well and exosomes *IN VIVO:* i.n. 10^7^ particles/10 μL × 2	Overcome multiple drug resistance in cancer cells	[49]
MDAs, hUVECs, hMSCs and hESCs	Porphyrins of different hydrophobicities	200 Ω, 500 μF, 200 mV, and pulse time of 20–30 ms	MDA-MB231	20,000 cells per well and EVs diluted 1:2 from the Stock solution (1.5 mg/mL of Por)	Improve the cellular uptake and photodynamic effect of porphyrins	[55]
B16-F10	5 nm SPIONs	High voltage setting	The formulation was not tested with cells or animals	The formulation was not tested with cells or animals	Maximizing exosome colloidal stability	[56]
HeLa, HTB-177, CD14^+^ monocytes and CD14^−^ lymphocytes	siRNA	0.150 kV/100 µF	HTB-177, CD14^+^ monocytes, and CD14^−^ lymphocytes	0.5 × 10^4^ cells per well and 30 μL of exosomes with siRNA at 2 μmol/mL	Deliver exogenous siRNA to monocytes and lymphocytes	[57]
**Sonication**						
RAW 264.7	Paclitaxel	20% amplitude, 6 cycles of 30 s on/off, 2 min pause, then incubated at 37 °C for 60 min	MDCK_WT_, MDCKMDR1 and 3LL-M27	5000 cells per well and exosomes *IN VIVO:* i.n. 10^7^ particles/10 μL × 2	Overcome multiple drug resistance in cancer cells	[49]
RAW 264.7	Enzyme catalase	Sonicated twice at 500 v, 2 kHz, 20% power, 6 cycles by 4 s pulse/2 s pause	Neuronal PC12 *IN VIVO*: C57BL/6 female mice	50,000 cells per well and EVs 230 µg total protein/mL *IN VIVO:* i.n. or i.v. 2.4 × 10^10^ EVs	Parkinson’s disease therapy	[51]
**Extrusion**						
RAW 264.7	Enzyme catalase	Extruded (x10 times) with 200 nm pores diameter	Neuronal PC12 *IN VIVO*: C57BL/6 female mice	50,000 cells per well and EVs 230 µg total protein/mL *IN VIVO:* i.n. or i.v. 2.4 × 10^10^ EVs	Parkinson’s disease therapy	[51]
MDAs, hUVECs, hMSCs and hESCs	Porphyrins of different hydrophobicities	Extruded at 42 °C (31 times) with 400 nm pore diameter	MDA-MB231	20,000 cells per well and EVs diluted 1:2 from the Stock solution (1.5 mg/mL of Por)	Improve the cellular uptake and photodynamic effect of porphyrins	[55]
**Freeze–Thaw**						
RAW 264.7	Enzyme catalase	Incubated for 30 min, then −80° C, then RT (three times)	Neuronal PC12 *IN VIVO*: C57BL/6 female mice	50,000 cells per well and EVs 230 µg total protein/mL *IN VIVO:* i.n. or i.v. 2.4 × 10^10^ EVs	Parkinson’s disease therapy	[51]
**Chemical-Based Transfection**						
MDAs, hUVECs, hMSCs and hESCs	Porphyrins of different hydrophobicities	Addition of 0.1 mg/mL saponin at RT for 10 min	MDA-MB231	20,000 cells per well and EVs diluted 1:2 from the Stock solution (1.5 mg/mL of Por)	Improve the cellular uptake and photodynamic effect of porphyrins	[55]
HeLa, HTB-177, CD14^+^ monocytes and CD14^−^ lymphocytes	siRNA	Addition of HiPerFect, then incubated for 10 min at RT	HTB-177, CD14^+^ monocytes, and CD14^−^ lymphocytes	0.5 × 10^4^ cells per well and 30 μL of exosomes with siRNA at 2 μmol/mL	Deliver exogenous siRNA to monocytes and lymphocytes	[57]
RAW 264.7	Enzyme catalase	Addition of 0.2% saponin, shaker for 20 min at RT, then incubated at RT for 18 hours	Neuronal PC12 *IN VIVO*: C57BL/6 female mice	50,000 cells per well and EVs 230 µg total protein/mL *IN VIVO:* i.n. or i.v. 2.4 × 10^10^ EVs	Parkinson’s disease therapy	[51]
HeLa and HT1080	siRNA	Addition of lipofectamine and incubated for 30 min at RT	HeLa and HT1080	0.5 × 10^6^ cells per well and varying amounts of exosomes (0–460 µg)	Deliver siRNA to recipient cells in vitro	[58]

**Table 2:** Applications of membrane functionalization through indirect methods.

Parental Cells	Functionalization	Cell Engineering Conditions	Recipient Cells	Treatment Conditions	Application	Reference
HEK293	Tetraspanins (CD63, CD9, CD81)	Transfected at 40~60% confluency using plasmid DNA (1–2 µg/well) for 48 h with PureFection Transfection Reagent or FuGENE6 t.r.	HEK293	Cells at confluency of 80% and 50 µg of exosomes	Tracking, imaging and targeting drug delivery	[60]
GM-CSF	Lamp-2b fused to the neuron-specific RVG peptide	Transfected 4 days using 5 µg of pLamp2b and 5 µl of TransIT LT1 t.r.	C2C12 and Neuro2A *IN VIVO*: C57BL/6 mice	Exosomes (12 µg proteins) and 400 nanomoles of siRNA * IN VIVO: * i.v. 150 µg of exosomes	Delivering of siRNA to the brain in mice with a reduced immunogenicity	[61]
Immaturedendritic cells (imDCs)	Lamp2b fused to CRGDKGPDC	Transfected with the vector expressing iRGD-Lamp2b fusion proteins using Lipofectamine 2000 t.r.	MDA-MB-231 *IN VIVO*: BALB/c nude mice	2 mM Dox-loaded exosomes *IN VIVO*: i.v. EVs 3mg/kg Dox loaded exosomes	Targeted tumour therapy	[62]
Neuro2A	GPI	Transfected with pLNCX-DAF-R2 or pLNCX-DAF-EGa1 using TransIT 2020 t.r.	Neuro2A, HeLa, and A431	40,000 cells per well or cells at a confluency of 80–90% and EVs at 5 µg/mL	Promoting tumor cell targeting	[63]
HEK293	GE11 or EGF	Transfected with pDisplay encoding GE11 or EGF using FuGENE HD t.r.	HCC70 HCC1954 MCF-7 *IN VIVO:* RAG2^–/–^ mice	1 × 10^5^ breast cancer cells and 1 µg of exosomes *IN VIVO*: i.v. 1 µg of exosomes, once per week for 4 weeks	Delivering of antitumor microRNA to EGFR-expressing breast cancer cells	[64]
BT474, SKBR3, HER2+, JAWSII DCs, 4T1-HER2, and bmDCs	CEA and HER2 coupled to the C1C2 domain of lactadherin	Transfected with p6mLC1C2 containing either human CEA (nt 1-2025) or human HER2/neu (nt 1-1953)	*IN VIVO*: C57BL/6J and BALB/c mice, hCEA or HER2 transgenic mice	*IN VIVO*: 2.6 × 10^10^ or 5.2 × 10^9^ or 1.05 × 10^9^ viral particles	Increasing vaccine potency	[65]
HEK293-F, E6, and CT26	PSA and PAP coupled to the C1C2 domain of lactadherin	Transfected with pPSA/Zeo, pPSA-C1C2/Zeo, pPAP/Hygro, or pPAP-C1C2/Hygro using Lipofectamine LTX reagent and PLUS Reagent	*IN VIVO*: Male BALB/c or C57BL/6 mice	*IN VIVO*: 5E7 TCID50 of the MVA-BN-PRO viral vectors once every 2 weeks for a total of three treatments	Targeting of tumor antigens to improve antigen immunogenicity and therapeutic efficacy	[66]
DCs	C1C2 domain of lactadherin	Transfected with modified p6mLC1C2 or pcDNA6-Myc/His using Fugene 6 t.r.	*IN VIVO*: Balb/C mice	*IN VIVO*: six inoculums of YAC exosomes with HLA-A2 or five inoculums of YAC/HLA-A2 exosomes with pMAGE-A3	Usage of antibodies against tumor biomarkers to attach the drug target candidates	[67]
THP-1	RGD- DSPE-PEG and/or DSPE-PEG-SH	Incubated with DSPE-PEG-SH and/or DSPE-PEG-RGD for 2 days	MCF-7 and HeLa *IN VIVO*: tumor-bearing mouse	4 × 10^5^ cells/mL and 100 µL per well of 50 µg/mL exosomes *IN VIVO*: i.v. 200 µL of exosomes at 5 mg/mL	Active targeted chemo-photothermal synergistic tumor therapy	[68]
THP-1	DSPE-PEG-biotin and/or DSPE-PEG-FA	Incubated with DSPE-PEG-biotin and/or DSPE-PEG-folate for 2 days	HeLa *IN VIVO*: C57BL/6 mice	40 μg/mL of EVs *IN VIVO:* i.v. EVs with a total of 1.16 mg iron	Rapid isolation and enhanced tumor targeting	[69]
Cal 27 cells	DSPE-PEG-biotin and DSPE-PEG-folate	Incubated with DSPE-PEG-biotin and DSPE-PEG-folate	MDA-MB-231 *IN VIVO*: BALB/C mice	Series of dose and concentration *IN VIVO*: 18–22 g of EVs via the tail vein	Enhanced target and synergistic therapy for breast cancer	[70]
HUVECs	DSPE-PEG-biotin (to then attach SA-QDs)	Cultured with DSPE-PEG-biotin for several days and then incubated with SA-QDs	EPCs *IN VIVO*: nude mice bearing A2058 xenografts	Short-term incubation *IN VIVO: * injection	Antitumor siRNA delivery	[71]
HUVECs	DSPE-PEG-biotin and SA-FITC	Incubated in modified medium containing 40 µg/mL DSPE-PEG-biotin for several days	HepG2 and 3T3 fibroblast *IN VIVO*: cervical cancer-bearing male BALB/c mice	5 × 10^3^ cells per well and 0, 10, 40, 80, 100, and 200 mg/mL of exosomes *IN VIVO:* exosomes at 5 mg/mL, 200µL per mice	Active targeted drug delivery to tumor cells	[72]
HEK 293T cells	GlucB with sshBirA to conjugate streptavidin–Alexa 680	Transduced with lentivirus vectors, CSCW-Gluc-IRES-GFP or CSCW-GlucB-IRES-GFP, then infection with CSCW-sshBirA-IRES-mCherry lentiviruses	*IN VIVO*: athymic nude mice spiked with EV-GlucB	*IN VIVO*: injected with a bolus of 100 μg EV-GlucB via retro-orbital vein or via tail vein	Multimodal imaging in vivo, as well as monitoring of EV levels in the organs and biofluids	[73]
B16BL6	Streptavidin–lactadherin and biotinylated GALA	4 × 10^6^ cells per dish transfected with the plasmid vector pCMVSAV−LA	MHC class I molecules of DCs	5 × 10^4^ cells per well and exosomes (1 μg of protein) diluted in 0.1 mL of Opti-MEM	Efficient cytosolic delivery of exosomal tumor antigens	[74]

**Table 3:** Applications of the direct methods and graphical abstracts from the references.

Parental Cells	Functionalization	Functionalization Step	Recipient Cells	Treatment Conditions	Application	Reference
**Covalent**						
PC12 cells	TAMRA-NHS	200 µL of Exos added to 1 mL 0.1 M sodium bicarbonate with 100mg TAMRA-NHS	PC12 cells	1 × 10^8^ cells and 100 µL of exosome solutions	Visualization of cellular uptake and intracellular trafficking of exosomes	[85]
4T1 cells	Alkyne groups conjugated with azide-fluor 545	80 μg of exosomes in PBS, Cu (II) sulfate pentahydrate, 1.44 M l-ascorbic acid, and bathophenanthrolinedisulfonic acid disodium salt trihydrate	4T1 cells	Cells at a confluency of 75% and 5 μg of exosomes in 100 μL RPMI	Surface functionalization of exosomes	[86]
Neuro2A and platelets	EGFR conjugated to DMPE-PEG derivatives	Conjugation in a 8.6:1000 molar ratio of nanobody/DMPE-PEG-maleimide micelles and then mixed with EVs	A431 and Neuro2A *IN VIVO*: Crl:NU-Foxn1nu mice with human tumor xenografts	3 × 10^4^ cells per well and 8 µg/mL of EVs *IN VIVO:* i.v. of 2.5 µg of EVs in 100 µL PBS	Enhancing cell specificity and circulation time of EVs	[87]
Bovine serum	DSPE and chemical conjugation by NHS-PEG	Physical: DSPE-PEG-biotin mixed with the EXOs (500 µg in PBS) Chemical: NHS-PEG-biotin reacted with the primary amines (500 nmol) on the EXOs	RAW264.7, DC2.4, and NIH3T3 *IN VIVO*: mice	6 × 10^5^ or 4 × 10^5^ cells per well and EXOs at an ICG concentration of 5.8 µg per well *IN VIVO*: s.i. at a DiI dose of 1.52 µg/kg	Efficient delivery of immune stimulators and antigens to the lymph nodes in vivo	[88]
RAW 264.7 cells and BMM from C57BL/6 mice	DSPE-PEG or DSPE-PEG-AA	Addition of DSPE-PEG or DSPE-PEG-AA at 50 μg/mL	*IN VIVO*: C57BL/6 with induced pulmonary metastases	*IN VIVO:* i.v. injected with the exos at 10^8^ particles/100 μL, *n* = 4 per group	Targeted paclitaxel delivery to pulmonary metastases	[89]
HEK293T cells	FA, PSMA RNA aptamer, and EGFR RNA aptamer conjugated to 3WJ	Cholesterol-triethylene glycol was conjugated into the arrow-tail of the pRNA-3WJ to promote the anchorage of the 3WJ onto the EV membrane	MDA-MB-231, KB, LNCaP (PSMA+), PC3 (PSMA–) *IN VIVO*: KB xenograft mice model	Incubation with cells *IN VIVO*: 1 dose of equivalent 0.5 mg siRNA/kg every 3 days for a total of 6 doses	Control RNA loading and ligand display on EVs for cancer regression	[90]
RAW 264.7	NRP-1-targeted peptide RGE	Surface modification with sulfo-NHS that can react with azide-modified RGE peptide, using salts and copper as catalyst	U251 and Bel-7404 *IN VIVO*: orthotopic glioma-bearing BALB/c nude mice	Cells and exos at the equivalent of 15 µg/mL of Cur/SPIONS *IN VIVO*: i.v. of Cur/SPIONS at 800 µg/200 µg Exos/200 µL PBS	Facilitate simultaneous imaging and therapy of glioma in vitro and in vivo	[91]
** Non-Covalent **						
HeLa	Cationic lipid formulation, LTX, and GALA	20 μL LTX added to a solution of exosomes and 20 μL GALA and incubated for 20 min at room temperature	HeLa and (CHO)-K1	2 mL with 2 × 10^5^ cells and 20 μg/mL of exosomes	Enhancing cytosolic delivery of exosomes	[92]
RTCs	Superparamagnetic magnetite colloidal nanocrystal clusters	1 mL of serum incubated with 200 µL of M-Tfs solution for 4 h at 4 °C	H22 cells *IN VIVO*: Kunming mice bearing a subcutaneous H22 cancer	0.1 mg/mL of exos in a simulated blood circulation at 32.85 cm/s (artery), 14.60 cm/s (vein), and 0.05 cm/s (capillary)	Targeted drug delivery vehicle for cancer therapy with magnetic properties	[93]
Human serum and C2C12	Rhodamine-labelled M12-CP05, FITC-labelled NP41-CP05	CP05 (200 µg/mL) incubated with nickel beads, added into the precentrifuged serum (200 µL), and incubated for 30 min at 4 °C under rotation	*IN VIVO*: dystrophin-deficient and immunodeficient nude mice and C57BL/6 mice	*IN VIVO*: i.m.1 or 2 µg of EXOs, i.v. EXOs in 100 µL of saline solution	Enabling targeting, cargo loading, and capture of exosomes from diverse origins	[94]
4T1, MCF-7, and PC3	DiR labelling	5µL of DIR, at a concentration of 220 µg/mL in ethanol, was mixed with 220 µg exosomes or liposomes in 100 µL PBS for 1 hour	*IN VIVO*: Balb/c, nude, and NOD.CB17- Prkdcscid/J mice with either 4T1 cells or PC3 cells	*IN VIVO*: i.v. 60 µg DIR-labeled exosomes in 200 µL PBS or i.t. 60 µg of DIR-labeled exosomes in 50 µL PBS	Biodistribution and delivery efficiency of unmodified tumor-derived exosomes	[95]
**Glycosylation**						
MLP29	Neuraminidase	Surface glycosylation of the EVs was manipulated by treatment with neuraminidase to remove the terminal residues of sialic acid	*IN VIVO*: wild-type mice	*IN VIVO*: i.v. of the EVs	Modification of the glycosylation of EVs to alter their biodistribution in vivo	[96]
U87 and GBM8	Glycosylation and insertion of targeting ligand to DC-SIGN	Treated with a pan-sialic acid hydrolase Neuraminidase for 30 min at 37 °C and/or incubated with palmitoyl-LewisY while vortexing for 10 min	MoDCs	500,000 cells incubated with EVs for 45 min on ice to allow receptor binding	Enhancing receptor-mediated targeting of dendritic cells	[97]
HEK293FT	Glycosylation of targeting-peptide-Lamp2b fusion proteins	1.5 mL of 0.971 M sucrose was slowly pipetted underneath the 8.5 ml of exosome solution	HEK293FT and Neuro2A	Cells at 50% confluency and EVs for 2 h at 37 °C	Stabilization of exosome-targeting peptides	[98]
**Hybridization**						
HEK293FT	CRISPR/CRISPR-associated protein 9 (Cas9) system	Addition of the plasmid–liposome complex to exosomes and incubated at 37 °C for 12 h in a volume ratio of 1:2	MSCs	Incubation with cells at 90% of confluency	Efficiently encapsulate large plasmids and be endocytosed in MSCs	[99]
RAW 264.7, CMS7-wt, and CMS7-HE	DOPC, DOPS, DOTAP, and DOPS/PEG-DSPE	Exosomes (300 μg/mL, protein) mixed with 100 μM liposomes in a volume ratio of 1:1 and then several freeze–thaw cycles	HeLa cells	4.5 μg protein in exosome incubated with 1 × 10^5^ HeLa cells for 4 h at 37 °C	Control and modify the performance of exosomal nanocarriers	[100]
HUVECs and MSCs	Phosphatidylcholine, phosphatidylethanolaminein, and PEG	Liposomes and EVs were mixed at 40 °C in a total volume of 40−200 μL (2 × 10^10^ or 2 × 10^11^ objects); liposome/EV ratio of 1:1, 1:9, or 9:1 in PBS. PEG was added at 5−30% (*w*/*v*)	THP1-derived macrophages and CT26	100,000 cells per well and hybrid EVs containing 1 mol % of DiR, cells, and 400 μL of mTHPC-loaded hybrid EVs or (3D) 500 cells and mTHPC-loaded hybrid EVs	Design of personalized biogenic drug delivery systems	[101]
J774A.1	L-a-phosphatidylcholine and cholesterol	EVs (200 µg protein) used to hydrate the dry 1000 µg of lipid film in a final volume of 1 mL; then, the solution was extruded through 400 and 200 nm polycarbonate membrane filter	K7M2, 4T1, and NIH/3T3	10,000–20,000 cells and 4 mL of 50 µg/mL of hybrid EVs at 37 °C for 3 h or 48 h	Tumor targeted drug delivery	[102]

**Table 4:** Applications of the top-down methods.

Precursor Cells	Recipient Cells	Application	Reference
**Extrusion**			
U937 and RAW 264.7	TNF-α-treated HUVECs *IN VIVO*: colon adenocarcinoma-induced CT26 mouse	Targeted delivery of chemotherapeutic drugs	[112]
RAW 264.7 and HB1.F3	*IN VIVO*: male BALB/c mice	Radiolabelling of EVs with ^99m^Tc-HMPAO to understand in vivo distribution and behavior of exosomes	[116]
Murine mouse embryonic stem cell line D3	NH-3T3	Gene delivery of endogenous, precursor cell characteristic RNA (mOct ¾ and mNanog)	[117]
Murine mouse embryonic stem cell line D3	Primary murine skin fibroblasts from BL6/C57 mice	Investigate the ability of these nanovesicles to improve proliferation by treating cells with the nanovesicles	[118]
Non-tumorigenic epithelial MCF-10A cells	MCF-7 *IN VIVO*: BALB/C nu/nu mice	Evaluation of the EV biosafety and uptake efficiency for the delivery of CDK4 siRNA	[119]
MSCs	MDA-MB-231 *IN VIVO*: nude BALB/c mice	Targeted delivery of paclitaxel for cancer treatment	[120]
H19-OE lentiviral vector-transfected HEK293	HMEC-1 *IN VIVO*: diabetic rat model	Treatment of diabetic wounds through the delivery of LncRNA-H19	[121]
MIN6 and NIH3T3	*IN VIVO*: BALB/c and NSG mice	Facilitation of the differentiation of bone marrow cells to insulin-producing cells (β-cells)	[122]
Primary hepatocytes	Primary hepatocytes *IN VIVO*: two-thirds PH mouse model (C57Bl/6)	Promote hepatocyte proliferation and liver regeneration	[115]
ASCs	MLE-12 *IN VIVO*: C57BL/6 mice	Inhibition of emphysema trough increasing the proliferation rate of lung epithelial cells	[123]
MSCs	RAW 264.7 *IN VIVO*: wild-type mice C57BL/6	Treatment of sepsis by down-regulating the cytokine storm induced by bacterial outer membrane vesicles (OMVs) in mice	[124]
M1 macrophages	CT26 and BMDMs *IN VIVO*: CT26-bearing mice	Repolarize M2 tumor-associated macrophages (TAMs) to M1 macrophages that release pro-inflammatory cytokines and induce antitumor immune responses	[125]
Natural killer (NK) cells NK92-MI	D54, MDA-MB-231, CAL-62, and HepG2 *IN VIVO*: female BALB/c nude mice	Immunotherapeutic agent for treatment of cancer	[114]
**Microfluidics**			
Murine embryonic stem cells (ES-D3)	NIH 3T3	Exogenous material delivery (polystyrene beads)	[126]
Murine embryonic stem cell line-D3	NIH-3T3 fibroblasts	Gene delivery of RNAs, Oct ¾, and Nanog	[127]

**Table 5:** Applications of the bottom-up methods and graphical abstracts from the references.

Formulation	Recipient Cells	Application	Reference
PC:CHOL:DSPE-PEG:DSPE-PEG-MAL liposome coated with MHC Class I/ peptide complexes, anti-LFA1, anti-CD28, anti-CD27, anti-4-1BB, anti-CD40L, and T cell receptors in the form of Fab antibody regions	T cells *IN VIVO*: BALB/c mice	Targeted immunotherapy, inducing antigen-specific T cells responses	[128]
DOPC/SM/Chol/DOPS/DOPE at a molar ratio of 21/17.5/30/14/17.5 liposome with siRNA (siNC, FAM-siNC, and siVEGF)	A549 and HUVEC	Delivery of VEGF siRNA in a more efficient way and with less cytotoxicity	[129]
DOPC/SM/Chol/DOPS/DOPE at a molar ratio of 21/17.5/30/14/17.5 liposome integrated with connexin 43 (Cx43)	A549 and U87 MG	Delivery of siRNA	[130]
CH/PC/SM/Cer at a weight ratio of 0.9/1/0.4/0.03 functionalized with recombinant human integrin α6β4 protein, bovine serum albumin, and lysozyme	A549 *IN VIVO*: mice bearing lung cancers	Targeted delivery of therapeutic oligonucleotides to lung adenocarcinoma cells (microRNA-145 mimics)	[131]
Phosphatidylcholine, SM, ovine wool cholesterol, and DOGS-NTA in a weight ratio of 55:30:10:5 liposome bonded with histidine-tagged APO2L/TRAIL	*IN VIVO*: adult female NZW rabbits	Treatment of antigen-induced arthritis (AIA)	[132]
Phosphatidylcholine, sphingomyelin (SM), cholesterol, and DOGS-NTA-Ni liposome with rAPO2L/TRAIL	Jurkat clone E6.1, U937, U266, and MM.1S	Apoptosis-inducing ability of hematological tumors	[133]
Cremophor EL, PC, DOPE, and DC-Chol liposome conjugated with DEC205 monoclonal antibody	DCs	Development of antigen carriers for specific DC targeting	[134]
^*^ Membrane proteins derived from RBCs (containing high CD47 levels to inhibit phagocytosis) and MCF-7 cancer cells (containing specific adhesion proteins) integrated into synthetic phospholipidic bilayers	MCF-7, HeLa, and RAW264.7 *IN VIVO*: MCF-7 tumor-bearing nude mice	Higher tumor accumulation, lower interception, and better antitumor therapeutic effect	[135]
* Proteins derived from the leukocytes’ plasmalemma trough extrusion integrated into a synthetic phospholipid bilayer (DPPC, DSPC, and DOPC and cholesterol)	*IN VIVO*: BALB/C mice	Selective and effective delivery of dexamethasone to inflamed tissues, and reduced phlogosis in a localized model of inflammation	[136]
* Membrane proteins derived from leukocytes from human blood and immortalized J774 murine macrophages within the lipid bilayer of liposome-like nanovesicles (DPPC, DOPC, and cholesterol in a molar ratio of 4/3/3)	HUVECs *IN VIVO*: Balb/c mice	Avoidance of macrophage uptake and promoting the adhesion to inflamed endothelium	[137]

* In these works, the EV-like nanovesicles are obtained with a bottom-up technique, but they integrate cellular membrane fragments that are extracted from cells with a top-down approach.

## Abbreviations

i.v.	intra venous
i.n.	intra nasal
i.p.	intra peritoneal
i.d.	intra dermal
i.m.	intramuscular
i.t.	intra tumour
s.i.	subcutaneously injection
t.r.	transfecting reagent
